# Trends in the Rates of Extended-Spectrum-β-Lactamase-Producing *Enterobacterales* Isolated from Urine Cultures during the COVID-19 Pandemic in Ontario, Canada

**DOI:** 10.1128/spectrum.03124-22

**Published:** 2023-01-16

**Authors:** Mohammad R. Hasan, Yasmeen M. Vincent, Daniela Leto, Huda Almohri

**Affiliations:** a Medical and Scientific Department, LifeLabs, Toronto, Ontario, Canada; b Department of Pathology and Molecular Medicine, McMaster University, Hamilton, Ontario, Canada; Health Canada

**Keywords:** COVID-19, *Escherichia coli*, extended-spectrum β-lactamase, *Klebsiella pneumoniae*, urine culture

## Abstract

Worldwide, extended-spectrum β-lactamase (ESBL) rates are increasing at an alarming level with increasing rates of health care exposures, international travel, and antibiotic usage. In this study, we investigated whether enhanced social isolation, travel restrictions, and the reduced use of antibiotics in Ontario, Canada during coronavirus disease 2019 (COVID-19) pandemic had an impact on ESBL rates in urine cultures collected from the community and long-term-care (LTC) facilities across the province. Data from a total of 8.6 million urine cultures performed at LifeLabs Ontario from 2016 to 2021 were utilized for analysis. ESBL-producing Escherichia coli (ESBL Escherichia coli) and ESBL Klebsiella pneumoniae were identified using standard operating procedures. Data trends were estimated by interrupted time series (ITS) regression analysis. Among 2.3 million positive urine cultures, 48.9% and 7.2% grew E. coli and K. pneumoniae, of which 5.8% and 3.3% produced ESBLs, respectively. While the overall rate of ESBL isolation was higher in the pandemic period than in the prepandemic period, by ITS regression analysis of the monthly rates of ESBL isolation, decreasing trends were noted for ESBL E. coli in both the community and LTC facilities and for ESBL K. pneumoniae in the community. The ESBL K. pneumoniae rates in LTC facilities continued to increase throughout the COVID-19 period. By subgroup analysis for different genders, age groups, and local health integration network (LHIN) units, similar trends were seen in most cases (*P < *0.05), except for a few densely populated LHINs where rate changes were not statistically significant.

**IMPORTANCE** Community-onset urinary tract infections (UTIs) caused by ESBL-producing *Enterobacterales*, particularly E. coli and K. pneumoniae, are a major public health concern. In this study, we assessed the impact of COVID-19 on ESBL rates in urine cultures in Ontario, Canada. Our results show the recent epidemiology of ESBL-producing *Enterobacterales* in urine cultures from both the community and LTC facilities in Ontario, Canada, and the impact of COVID-19 restrictions on ESBL trends for the entire province as well as different subgroups of the population based on demographic and geographic characteristics. Our results may have important public health implications in the context of the gradual easing of COVID-19 restrictions.

## INTRODUCTION

Extended-spectrum β-lactamases (ESBLs) are a family of genetically diverse β-lactamases that confer resistance to most β-lactam antibiotics, including penicillin, broad-spectrum cephalosporins, and monobactams, but are inactive against cephamycin and carbapenem antibiotics. Over the last few decades, ESBLs have evolved with new mechanisms of resistance and transmission, leading to their widespread distribution among *Enterobacterales* isolated from both hospital- and community-acquired infections (1). The original TEM- and SHV-type ESBLs have been gradually replaced by the CTX-M family ESBLs, particularly among Escherichia coli and Klebsiella pneumoniae isolates. The rapid proliferation of CTX-M-type ESBLs was facilitated by their mode of transmission via plasmids or other mobile genetic elements and by the clonal expansion of some highly successful lineages of E. coli (e.g., sequence type 131 [ST131] and ST405) and K. pneumoniae (e.g., clonal complex 11 [CC11] and ST147) as the vehicles for ESBLs (1–4).

Currently, an estimated 1.5 billion people in the world are colonized with ESBL-producing *Enterobacterales*, with major burdens on developing countries (2). However, ESBL rates are increasing in developed countries as well. For example, at U.S. hospitals, the proportion of ESBL producers among urinary E. coli isolates increased from 7.8% in 2010 to 18.3% in 2014. On the other hand, 16.3% of K. pneumoniae clinical isolates collected between 2011 and 2013 were ESBL producers (2, 5, 6). In a nationwide surveillance study, the proportions of ESBL-producing E. coli (ESBL E. coli) and ESBL K. pneumoniae strains isolated in Canadian hospitals during the period from 2007 to 2016 increased from 3.4% to 11.1% and from 1.3% to 9.7%, respectively (7). In parallel, ESBL rates are also increasing in the community, as reflected in the 5% annual increase in fecal carriage and the increasing rates of infections caused by ESBL-producing *Enterobacterales* (4, 8).

While the gastrointestinal tract is the main reservoir for ESBL-producing organisms, colonization with such organisms significantly increases the risk of ESBL-associated infections in other body sites, including the urinary tract, intraabdominal cavity, respiratory tract, and bloodstream (9). The risk factors for ESBL infections include health care exposures, antibiotic usage in humans and livestock, social and household contacts, and international travel, particularly to Asian countries (10–12), most of which were somewhat interrupted by the ongoing pandemic of coronavirus disease 2019 (COVID-19). In a recent study from Canada, the national rate of community antibiotic dispensing was reported to be decreased by 26.5% during the first 8 months of the COVID-19 pandemic (13). However, the implications of such a decrease in antibiotic prescriptions for the prevalence of antimicrobial resistance among bacteria isolated from different types of infections remained unknown. In this study, we have conducted a retrospective, observational study to compare the trends in the monthly rates of isolation of ESBL-producing E. coli and K. pneumoniae from urine cultures during and before the COVID-19 pandemic in Ontario, Canada, using segmented time series regression analysis.

## RESULTS

The province of Ontario has a total population of about 15 million people based on the most recent estimates (14). From January 2016 until the end of 2021, a total of 8,652,381 urine cultures from patients in the community and 456 long-term-care (LTC) facilities were performed in the microbiology laboratories of LifeLabs Ontario, of which 6,580,503 (76.1%) were reported as negative or insignificant (low colony counts or mixed organisms suggesting contamination). A total of 2,271,884 bacterial or yeast isolates were reported for the remaining cultures, of which 1,110,375 (48.9%) were E. coli and 162,982 (7.2%) were K. pneumoniae. Of all E. coli isolates, 65,734 (5.8%) produced ESBLs, and of all K. pneumoniae isolates, 5,665 (3.3%) produced ESBLs. The rates of ESBL production by both E. coli and K. pneumoniae were much higher (*P < *2.2E−16) for patients in LTC facilities than for those in the community. For example, 17.6% of E. coli strains isolated from urine samples from LTC patients showed the ESBL phenotype, as opposed to 5.2% of those in the community. Similarly, a significantly higher (*P < *2.2E−16) proportion of K. pneumoniae strains (7%) isolated from urine samples from LTC patients showed the ESBL phenotype than in the community (3.1%).

The rates of isolation of ESBLs from urine culture were also much higher (*P < *2.2E−16) in male patients than in female patients from communities as well as LTC facilities. ESBL E. coli rates were 11.3% and 26.6% in males compared to 4.5% and 16.0% in females in communities and LTC facilities, respectively. By age group, the ESBL E. coli rate in the community was much higher (*P < *2.2E−16) in patients over 60 years of age (6.7%) than in patients under 18 years of age (3.2%) and those 18 to 59 years of age (3.7%). Similarly, ESBL K. pneumoniae rates in males were 6.1% and 12.2% (significantly higher at a *P* value of <2.2E−16) compared to only 2.6% and 5.2% in females in the community and LTC facilities, respectively. By age group, the ESBL K. pneumoniae rate in the community was slightly but significantly (*P < *6.7E−13) higher in patients over 60 years of age (3.3%) than in patients 18 to 59 years of age (2.5%).

Among the 14 local health integration network (LHIN) units, the rates of both ESBL E. coli and K. pneumoniae in urine cultures from the community were at the higher end in the Central West (9.1% ESBL E. coli and 3.9% ESBL K. pneumoniae) and the Toronto Central (6.1% ESBL E. coli and 4% ESBL K. pneumoniae) LHINs. For LTC facilities, both ESBL E. coli (28.5%) and ESBL K. pneumoniae (14.1%) were isolated at the highest rates in the North West LHIN. The rates of isolation of both ESBL E. coli and ESBL K. pneumoniae in the community were at the lower end in the LHINs located in the eastern and northern regions of the province, such as North East, South East, and North Simcoe Muskoka. ESBL E. coli and ESBL K. pneumoniae rates were the lowest in LTC facilities within the South West and North Simcoe Muskoka LHINs, respectively.

By calendar year, the rates of E. coli and K. pneumoniae isolates producing the ESBL phenotype gradually increased since the beginning of 2016 but showed a declining trend from the second quarter of 2020, after COVID-19-related travel and social restrictions were implemented by the province of Ontario. The only exception was the rate of ESBL K. pneumoniae strains isolated at LTC facilities, which showed a persistent increase throughout the pandemic from the second quarter of 2018 (see Fig. S1 in the supplemental material). The drop in community cases of ESBL-producing E. coli and K. pneumoniae in 2018 reflects the lack of data during the third and fourth quarters of 2018 when testing for ESBL in the community was temporarily paused at LifeLabs following Clinical and Laboratory Standards Institute (CLSI) recommendations to use updated MIC breakpoints for β-lactam antibiotics without the need for an ESBL confirmatory test (see Materials and Methods) (15). ESBL confirmatory testing at LifeLabs was reinstated in 2020 for infection prevention and epidemiological purposes.

The overall rate of isolation of ESBL E. coli in urine cultures from the community for the province of Ontario during (pre-COVID-19 period) or before the implementation of COVID-19-associated travel and social restrictions was 4.8%, compared to 6.3% during the COVID-19 period (Table 1). Similar increases in rates were seen during the COVID-19 period for ESBL K. pneumoniae in the community as well as for both organisms in LTC facilities (Tables 1 and 2). When the E. coli ESBL data were broken down by gender, by age group, and by LHIN, an overall increase in the ESBL rate during the COVID-19 period was seen in almost all cases except for LTC patients in some LHINs such as Central East, Central West, Champlain, Erie St. Clair, and Mississauga Halton, where the rates of ESBL E. coli were slightly decreased (Table 1). For ESBL K. pneumoniae in the community, the rates increased during the COVID-19 period irrespective of gender, age group, and LHIN, except that the rates did not significantly change (*P* = 0.51) in children under 18 years of age. For ESBL K. pneumoniae in LTC facilities, the rates increased during the COVID-19 period irrespective of gender and age group. However, rate changes in different LHINs were variable, and the numbers were very low for some LHINs (Table 2).

**TABLE 1:** Epidemiological characteristics of patients with community-acquired or LTC-acquired bacteriuria with ESBL E. coli in Ontario, Canada, from 2016 to 2021

Characteristic	Community					Long-term care				
	Pre-COVID-19 period (Jan 2016 to Mar 2020)		COVID-19 period (Apr 2020 to Dec 2021)			Pre-COVID-19 period (Jan 2016 to Mar 2020)		COVID-19 period (Apr 2020 to Dec 2021)		
	No. of E. coli isolates	No. (%) of ESBL E. coli isolates	No. of E. coli isolates	No. (%) of ESBL E. coli isolates	*P* value	No. of E. coli isolates	No. (%) of ESBL E. coli isolates	No. of E. coli isolates	No. (%) of ESBL E. coli isolates	*P* value
Ontario total	804,157	38,734 (4.8)	237,453	14,909 (6.3)	1.8E−176	50,618	8,586 (17)	18,147	3,505 (19.3)	9.24E−13
Age (yrs)										
0 to <18	46,068	1,387 (3)	13,531	529 (3.9)	1.88E−07					
18 to <60	383,932	13,060 (3.4)	97,607	4,833 (5)	1.2E−115	1,359	233 (17.1)	469	105 (22.4)	0.011674
≥60	374,157	24,287 (6.5)	126,315	9,547 (7.6)	5.61E−39	49,254	8,352 (17)	17,676	3,400 (19.2)	8.55E−12
Gender										
Female	732,242	31,078 (4.2)	212,645	11,662 (5.5)	1.3E−129	43,363	6,713 (15.5)	15,357	2,710 (17.6)	3.31E−10
Male	71,915	7,656 (10.6)	24,808	3,247 (13.1)	9.64E−26	7,255	1,873 (25.8)	2,790	795 (28.5)	0.006492
LHIN										
Central	120,366	6,476 (5.4)	30,122	2,124 (7.1)	5.45E−29	4,478	772 (17.2)	1,413	262 (18.5)	0.261894
Central East	85,145	4,378 (5.1)	23,376	1,582 (6.8)	4.27E−22	4,957	748 (15.1)	1,958	279 (14.2)	0.375874
Central West	44,096	3,862 (8.8)	11,482	1,204 (10.5)	1.01E−08	1,023	291 (28.4)	267	59 (22.1)	0.03775
Champlain	33,236	1,121 (3.4)	11,189	630 (5.6)	2.52E−26	2,169	535 (24.7)	659	154 (23.4)	0.496971
Erie St. Clair	19,968	614 (3.1)	8,051	381 (4.7)	1.17E−11	2,556	516 (20.2)	1,085	204 (18.8)	0.336888
Hamilton Niagara Haldimand Brant	112,656	5,719 (5.1)	36,405	2,196 (6)	1.56E−12	9,261	1,515 (16.4)	3,356	680 (20.3)	3.21E−07
Mississauga Halton	73,928	4,659 (6.3)	20,427	1,616 (7.9)	3.09E−16	2,499	590 (23.6)	926	214 (23.1)	0.759448
North East	27,260	646 (2.4)	9,560	352 (3.7)	1.06E−11	5,065	995 (19.6)	2,194	607 (27.7)	3.79E−14
North Simcoe Muskoka	31,450	911 (2.9)	9,467	415 (4.4)	7.88E−13	1,836	260 (14.2)	537	85 (15.8)	0.334927
North West	12,721	481 (3.8)	4,490	210 (4.7)	0.008561	1,629	452 (27.7)	516	159 (30.8)	0.178586
South East	44,520	1,116 (2.5)	13,517	555 (4.1)	2.07E−22	3,576	555 (15.5)	1,210	201 (16.6)	0.368208
South West	51,930	1,544 (3)	17,283	895 (5.2)	3.06E−42	7,224	713 (9.9)	2,517	325 (12.9)	2.04E−05
Toronto Central	80,945	4,563 (5.6)	22,577	1,729 (7.7)	2.62E−29	1,923	362 (18.8)	676	136 (20.1)	0.46229
Waterloo Wellington	65,936	2,644 (4)	19,507	1,020 (5.2)	1.56E−13	2,422	282 (11.6)	833	140 (16.8)	0.00013

**TABLE 2:** Epidemiological characteristics of patients with community-acquired or LTC-acquired bacteriuria with ESBL K. pneumoniae in Ontario, Canada, from 2016 to 2021

Characteristic	Community					Long-term care				
	Pre-COVID-19 period (Jan 2016 to Mar 2020)		COVID-19 period (Apr 2020 to Dec 2021)			Pre-COVID-19 period (Jan 2016 to Mar 2020)		COVID-19 period (Apr 2020 to Dec 2021)		
	No. of K. pneumoniae isolates	No. (%) of ESBL K. pneumoniae isolates	No. of K. pneumoniae isolates	No. (%) of ESBL K. pneumoniae isolates	*P* value	No. of K. pneumoniae isolates	No. (%) of ESBL K. pneumoniae isolates	No. of K. pneumoniae isolates	No. (%) of ESBL K. pneumoniae isolates	*P* value
Ontario total	110,324	3,144 (2.8)	38,133	1,503 (3.8)	4.89E−26	10,348	611 (5.6)	4,177	407 (8.9)	2.33E−16
Age (yrs)										
0 to <18	2,194	64 (2.9)	658	16 (2.4)	0.508325					
18 to <60	34,707	789 (2.3)	9,952	343 (3.4)	5.22E−11	320	31 (9.7)	168	23 (13.7)	0.18047
≥60	76,567	2,291 (3)	29,026	1,144 (3.9)	8.37E−15	10,638	580 (5.5)	4,416	384 (8.7)	1.35E−13
Gender										
Female	99,578	2,376 (2.4)	33,747	1,055 (3.1)	1.16E−13	9,208	430 (4.7)	3,783	250 (6.6)	6.56E−06
Male	13,890	768 (5.5)	5,889	448 (7.6)	2.64E−08	1,751	181 (10.3)	801	157 (19.6)	1.49E−10
LHIN										
Central	15,911	517 (3.2)	4,778	168 (3.5)	0.366061	861	54 (6.3)	335	12 (3.6)	0.067358
Central East	11,917	280 (2.3)	4,009	137 (3.4)	0.00025	1,031	29 (2.8)	496	36 (7.3)	5.59E−05
Central West	5,598	208 (3.7)	1,650	73 (4.4)	0.190053	222	8 (3.6)	80	4 (5)	0.58355
Champlain	5,202	157 (3)	2,290	49 (2.1)	0.032208	511	41 (8)	172	31 (18)	0.000221
Erie St. Clair	3,225	51 (1.6)	1,403	45 (3.2)	0.000361	590	39 (6.6)	269	34 (12.6)	0.003294
Hamilton Niagara Haldimand Brant	16,869	570 (3.4)	6,544	238 (3.6)	0.331897	2,020	92 (4.6)	909	49 (5.4)	0.328105
Mississauga Halton	9,887	277 (2.8)	3,345	144 (4.3)	1.85E−05	678	28 (4.1)	231	3 (1.3)	0.040607
North East	4,349	69 (1.6)	1,678	91 (5.4)	1E−16	935	70 (7.5)	494	88 (17.8)	3.21E−09
North Simcoe Muskoka	4,812	74 (1.5)	1,630	55 (3.4)	4.78E−06	385	2 (0.5)	114	2 (1.8)	0.194021
North West	1,951	47 (2.4)	669	30 (4.5)	0.006097	308	17 (5.5)	160	49 (30.6)	1.34E−13
South East	6,796	96 (1.4)	2,268	66 (2.9)	3.15E−06	829	32 (3.9)	295	40 (13.6)	5.13E−09
South West	7,702	168 (2.2)	2,736	93 (3.4)	0.000457	1,556	116 (7.5)	573	34 (5.9)	0.223781
Toronto Central	10,784	385 (3.6)	3,745	197 (5.3)	5.51E−06	432	29 (6.7)	234	14 (6)	0.714375
Waterloo Wellington	8,465	245 (2.9)	2,891	117 (4)	0.002317	601	54 (9)	222	11 (5)	0.0571

**FIG 1:**
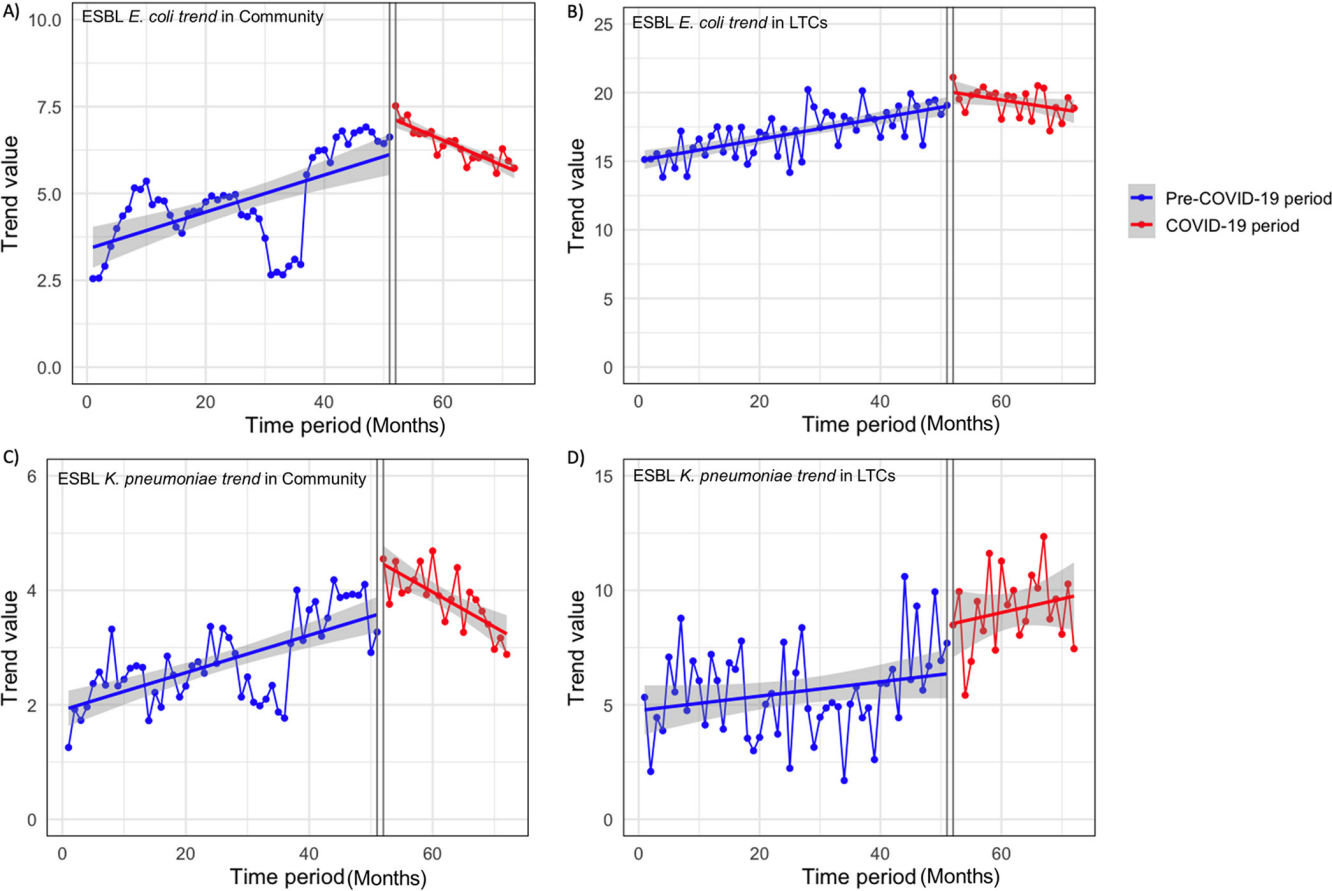
ESBL trends in urine cultures before and during the COVID-19 pandemic in Ontario, Canada. (A) ESBL E. coli trends in the community; (B) ESBL E. coli trends in LTC facilities; (C) ESBL K. pneumoniae trends in the community; (D) ESBL K. pneumoniae trends in LTC facilities. Dotted lines are predicted trends based on the seasonally adjusted regression model. Solid lines are deseasonalized trends. The time period is in months from January 2016 to December 2021.

**FIG 2:**
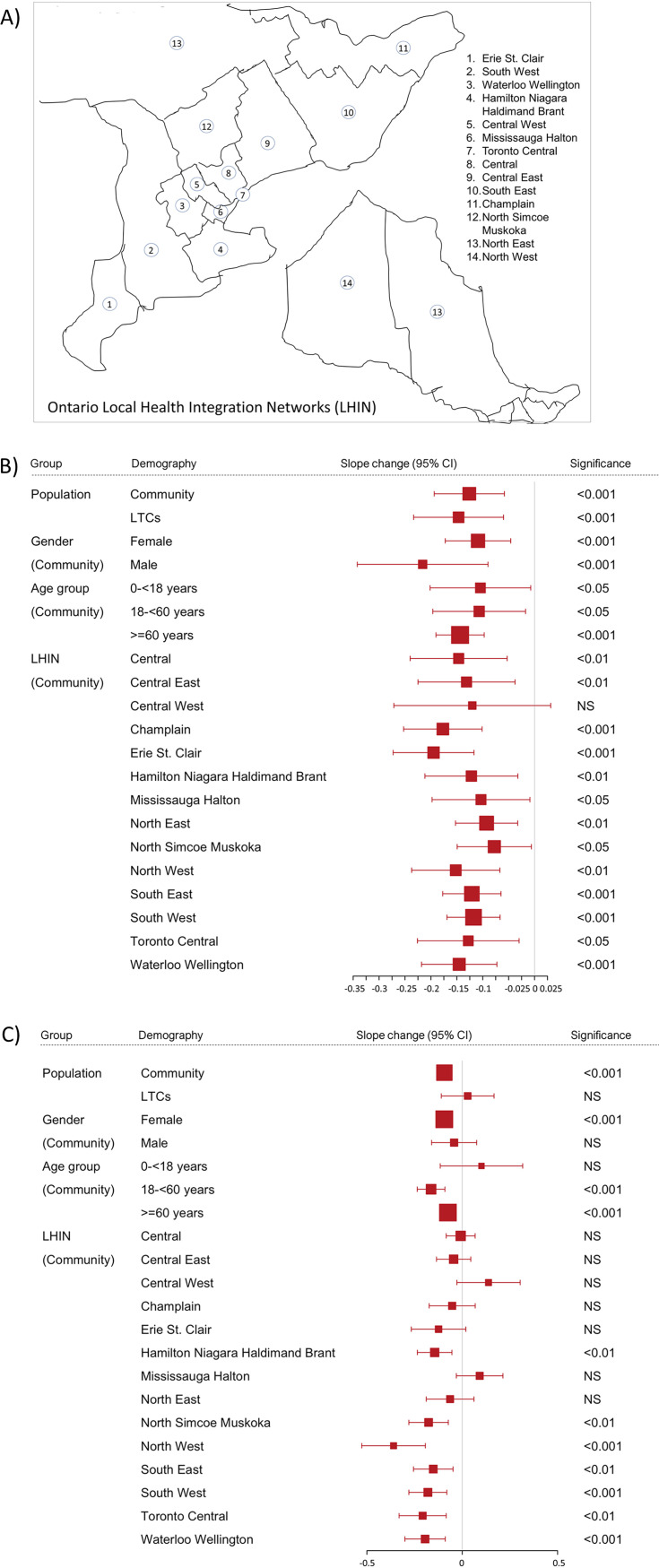
Changes in monthly rates of isolation of ESBL-producing *Enterobacterales* during the COVID-19 period. For each subgroup of the population, slope changes with confidence intervals and significance values were estimated from the deseasonalized time series of monthly ESBL rates by linear regression analysis as described in Materials and Methods. (A) Ontario LHIN map (created in Microsoft PowerPoint using the freeform drawing tool based on the Statistics Canada LHIN map, available at https://www150.statcan.gc.ca/n1/pub/82-402-x/2017001/maps-cartes/rm-cr08-eng.htm). (B) Slope changes for ESBL E. coli. (C) Slope changes for ESBL K. pneumoniae. NS, not significant.

While the overall ESBL rates were higher during the COVID-19 period than during the pre-COVID-19 period, yearly data on ESBL rates (Fig. S1) show a declining trend during the COVID-19 period. Therefore, we performed an interrupted time series (ITS) analysis of ESBL trends before and after the implementation of COVID-19-associated social and travel restrictions in Ontario, Canada. Trend values were calculated from monthly rates of ESBLs and, after adjustment for seasonality, were plotted against the number of months since the beginning of the study in January 2016 (Fig. 1). While before the pandemic, an upward trend in monthly ESBL rates was seen for both ESBL-producing E. coli and K. pneumoniae in urine cultures from both the community and LTC facilities, the monthly ESBL rates were trending downward in most cases starting in April 2020, soon after social and travel restrictions were implemented in Ontario because of COVID-19. Exceptionally, the rates of ESBL K. pneumoniae in LTC facilities kept increasing throughout the COVID-19 period. For ESBL E. coli, by linear regression analysis of deseasonalized, pre- and postintervention time series, the monthly ESBL rates during the COVID-19 period in the community and LTC facilities declined by 0.126% (95% confidence interval [CI], 0.058% to 0.194%) (*P *= 4.2E−04) and 0.147% (95% CI, 0.06% to 0.233%) (*P* = 0.00123), respectively (Fig. 2). On the other hand, for ESBL K. pneumoniae, the monthly ESBL rates during the COVID-19 period declined only in the community by 0.094% (95% CI, 0.056% to 0.132%) (*P* = 6.1E−06). The change in the monthly ESBL rates for K. pneumoniae was not significantly different in LTC facilities (*P* = 0.682).

Next, we assessed whether the changes in ESBL trends during the COVID-19 period were different for different genders, age groups, and geographical units in the community population only. We noted that the monthly rates of isolation of ESBL-producing E. coli in urine cultures decreased significantly irrespective of gender, age, and LHIN, except for only one LHIN (Central West) where the rate of decrease was not statistically significant. On the other hand, for ESBL K. pneumoniae, a significant reduction in the monthly rate was seen only for female gender, age groups over the age of 18 years, and 7 out of 14 LHINs.

**FIG 3:**
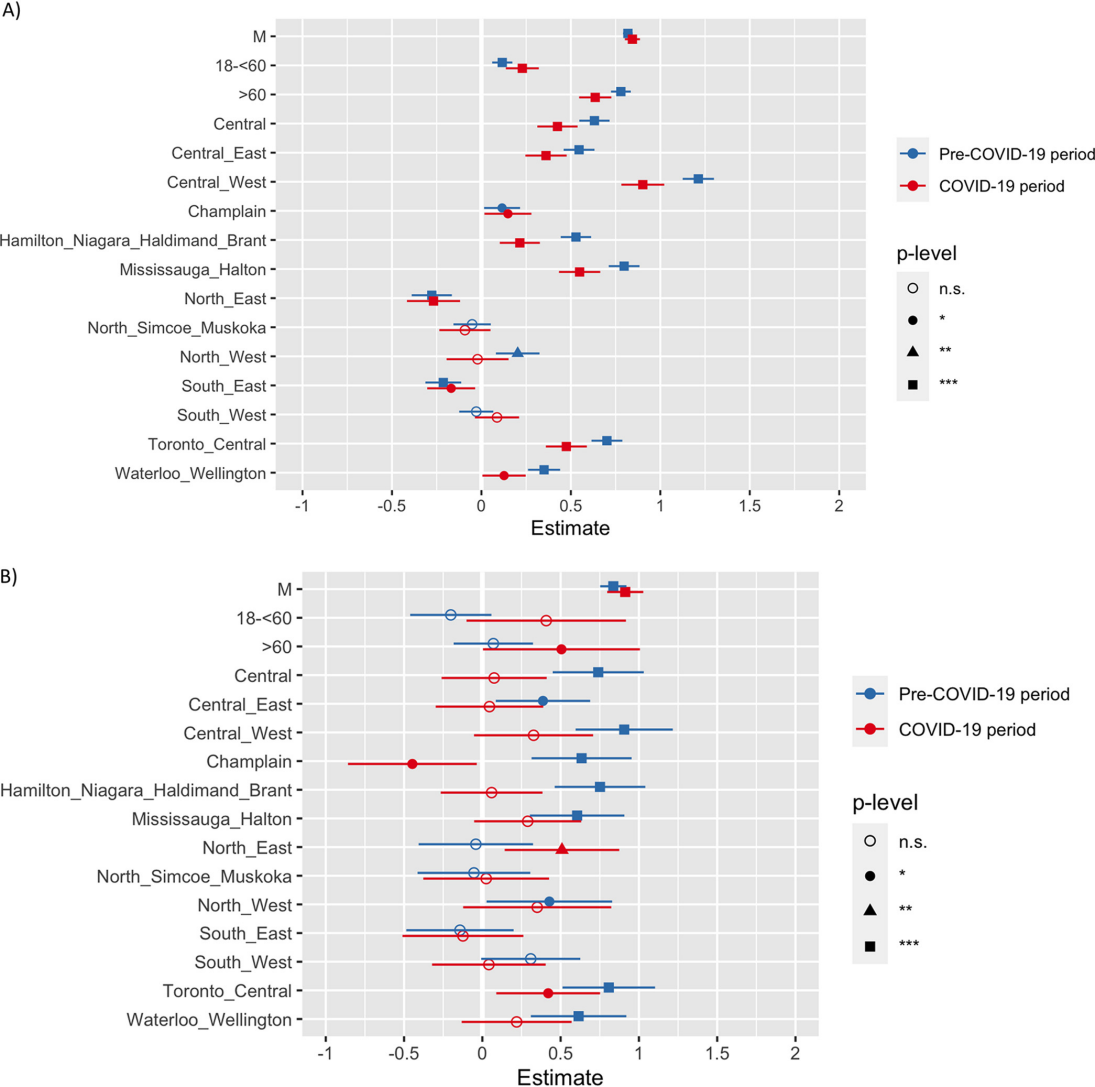
Association of ESBL-producing organisms in urine cultures with demographic and geographic factors during the COVID-19 pandemic compared to the pre-COVID-19 period. Multivariate regression models are adjusted for gender, age group (0 to <18, 18 to <60, and ≥60 years), and LHIN. (A) Association of ESBL E. coli in urine cultures with various demographic and geographic factors in the community. (B) Association of ESBL K. pneumoniae in urine cultures with various demographic and geographic factors in the community. M, male; n.s., not significant; *, *P* ≤ 0.05; **, *P* ≤ 0.01; ***, *P* ≤ 0.001.

Apart from the trends, we also compared the association of ESBL rates in urine cultures with patient demographic and geographic factors during the pre-COVID-19 period with that during the COVID-19 period by multivariate logistic regression analysis. For ESBL E. coli, the rates were most significantly (*P* < 0.001) positively correlated with male gender, an age of >60 years, and densely populated Central LHINs (Fig. 3). Although the direction of the association remained unchanged in these LHINs during the COVID-19 restriction period, the strength of the association (beta coefficient) decreased during the COVID-19 period. For ESBL K. pneumoniae, the rates remained highly correlated with male gender in both time periods. However, significant differences were noticed in the association between ESBL rates and an age of >60 years and between ESBL rates and centrally located LHINs. For example, the ESBL K. pneumoniae rates were not significantly associated with an age of >60 years in the pre-COVID-19 period, which has now turned significant during the COVID-19 period. Again, the level of significance for the ESBL association with several LHINs, including Central, Central West, Central East, Toronto Central, and Mississauga Halton, changed from significant to nonsignificant during the COVID-19 period. Exceptionally, the ESBL association changed from a positive to a negative direction in the Champlin LHIN during the COVID-19 period. On the other hand, the ESBL association with the Northeast LHIN significantly increased during the COVID-19 period.

## DISCUSSION

The impact of the COVID-19 pandemic on the prevalence of antimicrobial resistance has been a topic of much debate in recent years (16). On one front, it appeared likely that antimicrobial resistance rates would be higher because of the overutilization of antimicrobials for patients with COVID-19, with or without secondary infection, and because of the increased rate of hospitalization associated with COVID-19. Indeed, in the hospital setting, an increasing incidence of antimicrobial resistance has been reported in several studies (17). On the other front, predictions were made that antimicrobial resistance rates would decrease in the community because of social distancing, travel restrictions, and enhanced infection control and prevention practices. Consistently, in a French study conducted between January 2019 and December 2020, reductions in the rates of ESBL E. coli isolation from clinical samples from patients in primary care facilities and nursing homes were noted beginning in May 2020 compared to those before March 2020 (2.9% versus 3.1% [*P* < 0.001]) (18). While variation of such observations by country has been suggested, in Canada, based on a recently conducted ecological study, antibiotic prescriptions were significantly reduced during the first 8 months of the COVID-19 pandemic, an additional cause that may influence the prevalence of antimicrobial resistance in the community. To this end, we compared the trends of isolation of both ESBL E. coli and ESBL K. pneumoniae from urine samples 52 months before and 21 months after the declaration of COVID-19-related social restrictions, utilizing the urine culture data for the entire province of Ontario, Canada, from January 2016 to December 2021. The data represent patients from both the community and LTC facilities across the province.

The duration of the pre-COVID-19 period was chosen to be much longer than that of the COVID-19 period for the accurate determination of ESBL trends over time before COVID-19 and partly because some data were missing for the last two quarters of 2018. Based on monthly isolation rates, ESBL rates kept increasing from 2016 until the second quarter of 2020, just when travel and social restrictions were put into place because of COVID-19. Therefore, baseline ESBL rates were already very high early in 2020. As a result, the overall rates of ESBL isolation from urine cultures during the COVID-19 period were significantly higher than those during the pre-COVID-19 period (Tables 1 and 2). However, by ITS analysis, we have shown that there is a statistically significant, declining trend in the proportion of ESBL-producing E. coli and K. pneumoniae in urine cultures from community patients during the COVID-19 period (Fig. 1). On the other hand, in LTC facilities, a declining trend during the COVID-19 period was seen only in the case of ESBL E. coli but not in the case of ESBL K. pneumoniae. The reason for the different trend for ESBL K. pneumoniae in LTC facilities is unclear, but if outbreaks of ESBL K. pneumoniae occurred in one or more LTC facilities during the COVID-19 pandemic, this may explain the increasing trend (see Fig. S2 in the supplemental material). Although outbreaks of ESBL E. coli may have happened as well, ESBL K. pneumoniae trend values are particularly affected because of their low numbers in both periods. Notably, ESBL K. pneumoniae was never isolated from urine cultures from 238 of 456 LTC facilities (52%), as opposed to only 7 of 456 LTC facilities (1.5%) where E. coli ESBL was never isolated from urine cultures (data not shown).

The overall impact of COVID-19 restrictions on ESBL trends in Ontario is further supported by the fact that similar trends were seen in most subgroups of the population assessed based on gender, age group, and public health unit of the province (Fig. 2). However, in the case of ESBL K. pneumoniae, a few exceptions were noted. The slope change of the monthly ESBL K. pneumoniae rate for the 0- to 18-year age group was not statistically significant, but the results for this subgroup may have been affected by its small sample size. Also, unlike ESBL E. coli, slope changes were not statistically significant in many of the centrally located LHINs. Overall, it appears that ESBL trends continued to rise or decline less significantly in LHINs that are densely populated and ethnically diverse (19). Despite COVID-19 restrictions, there were no significant changes in the ESBL trends in the Central West LHIN, which represents a large population of immigrants from the Indian subcontinent, where the rate of carriage of ESBL-producing *Enterobacterales* is known to be very high (19–21).

Apart from the impact of COVID-19, recently published epidemiological data on ESBLs from either screening tests or clinical samples are limited for the province of Ontario. As such, our results provide a recent picture of ESBL epidemiology in Ontario. ESBL rates in urine cultures from LTC patients were much higher in our study than in those from community patients. The proportions of ESBL-producing E. coli strains isolated from urine cultures from LTC facilities were even higher than those reported for Canadian hospitals in 2016, suggesting a dramatic increase in such cases in recent years (Tables 1 and 2) (7). ESBL rates were also much higher in males than in females in both the community and LTC facilities. These results are consistent with the known epidemiology of ESBL-producing *Enterobacterales* in urine cultures, as reported previously (22). Also, the median age of males (71 years [interquartile range {IQR}, 58 to 81 years]) with ESBLs was higher than the median age of females (61 years [IQR, 38 to 77 years]). This may be due to a higher rate of catheter-related infections and/or prostatitis in this subpopulation requiring prolonged treatment with extended-spectrum β-lactam antibiotics. For ESBL E. coli, higher isolation rates are expectedly associated with older ages during both periods, but for ESBL K. pneumoniae, a significant association with an age of >60 years has been noted only recently, during the COVID-19 period.

Urinary tract infection (UTI) is one of the most common reasons for primary and emergency care visits in the community, and an increase in the rate of UTI caused by ESBL-producing *Enterobacterales* poses serious challenges for the treatment of these infections and increases the dependence on broad-spectrum, last-resort antibiotics such as carbapenems (23).

One major limitation of our study is that some ESBL data for Ontario are missing for the last two quarters of 2018. However, this is unlikely to affect the results of our study because data trends were calculated based on monthly ESBL rates starting in January 2016. Nevertheless, our results clearly show that ESBL trends went down, particularly in the community, since COVID-19 restrictions were implemented in Ontario, Canada. This may be related to the reduced use of antibiotics and social isolation or travel restrictions. Therefore, with the easing of COVID-19 restrictions in early 2022, it is likely that ESBL trends will rise again. Additionally, our results summarize the recent epidemiology of ESBL-producing *Enterobacterales* in urine cultures representing the entire province of Ontario. This may have important public health implications, including the need for enhanced surveillance programs and awareness in the community to prevent further increases in community-onset urinary tract infections caused by ESBL-producing organisms.

## MATERIALS AND METHODS

### Settings and data sources.

The study population included patients with symptomatic or asymptomatic bacteriuria acquired in the community (including primary care received in family physician clinics and walk-in clinics) or in long-term-care (LTC) facilities across the province of Ontario, Canada. Retrospective data from urine cultures performed by LifeLabs Medical Laboratories in Ontario from January 2016 to December 2021 were utilized in this study. Urine specimens for culture were collected from patients attending their family physicians or walk-in clinics or were collected at one of the 456 LTC facilities across the province. Unless otherwise stated, urine specimens were collected according to guidelines for the proper collection of midstream urine samples. Urine samples were transferred into tubes with preservatives once they were received at LifeLabs specimen collection centers. Retrospective, anonymous patient data and laboratory data were collected according to the LifeLabs code of ethics policy. Only limited patient data were collected, without any patient-identifying information. Data on age, gender, and location by local health integration network (LHIN) unit were retrieved only for patients with bacteriuria with ESBL- or non-ESBL-producing E. coli or K. pneumoniae from the laboratory information systems (LISs) using organism-specific queries. Patients were grouped into 3 age groups: (i) children 0 to <18 years of age, (ii) adults 18 to <60 years of age, and (iii) adults >60 years of age. The 14 LHINs dividing the entire province of Ontario are as follows: Central, Central East, Central West, Champlain, Erie St. Clair, Hamilton Niagara Haldimand Brant, Mississauga Halton, North East, North Simcoe Muskoka, North West, South East, South West, Toronto Central, and Waterloo Wellington.

### Urine culture and antibiotic susceptibility testing.

Urine cultures were performed at any of the five microbiology laboratories of LifeLabs Ontario located in Toronto, Mississauga, Bellville, Sudbury, or Thunder Bay depending on the geographical origin of the specimens. Both midstream urine and catheterized urine specimens were received. However, 98.8% of all specimens were midstream urine specimens. The collected urine samples were refrigerated and processed within 24 h of collection. For urine samples that may require >24 h for transport, samples were transferred to BD Vacutainer Plus C&S preservative tubes and processed within 48 h according to the manufacturer’s instructions. Culture and sensitivity determinations were performed according to standard procedures. All collection devices and laboratory procedures at LifeLabs were internally verified and validated prior to use according to CLSI standards (24, 25). Briefly, urine samples were plated in BBL CHROMagar orientation medium (BD) using 0.01-mL or 0.001-mL calibrated loops and incubated at 35°C for 18 to 24 h. Organisms were identified according to the color of the colonies. Further identification and susceptibility testing were done using the Vitek 2 system (bioMérieux). *Enterobacterales* producing the ESBL phenotype were identified based on Vitek 2 results. For isolates that were ESBL positive by Vitek 2 but were susceptible to a third-generation cephalosporin, ESBL phenotypes were further confirmed using a Kirby-Bauer (KB) disk diffusion susceptibility test protocol according to CLSI guidelines (24, 25). Antibiotic susceptibility testing (AST) results were recorded and reported for recognized uropathogens only if the colony counts were significant (>100 × 10^6^ CFU/L). However, the identification and AST results of bacteria with lower colony counts were reported for invasively collected specimens or at the discretion of a microbiologist. All E. coli and K. pneumoniae strains isolated from urine cultures were screened for ESBLs except during the last two quarters of 2018 when routine ESBL screening was stopped for samples from the community. Instead, antibiotic susceptibility test results were reported without an ESBL designation according to CLSI guidelines (15).

### Outcome measures and variables.

For interrupted time series analysis of ESBL trends, intervention was defined as the implementation of COVID-19 social restrictions, which were first declared in Ontario in March 2020. Therefore, the period from January 2016 to March 2020 was defined as the preintervention period (pre-COVID-19 period), and the period from April 2020 to December 2021 was defined as the postintervention period (COVID-19 period). Monthly rates of ESBL production by E. coli and K. pneumoniae strains isolated from urine cultures were the outcomes of the study. Independent variables were the time elapsed in months since the start of the study, the time elapsed in months since the intervention, and a dummy variable with the pre-COVID-19 period defined as “0” and the COVID-19 period defined as “1.” Sequential monthly data were available for a total of 51 months during the pre-COVID-19 period and a total of 21 months during the COVID-19 period. The monthly rates of ESBL production (percent) by E. coli and K. pneumoniae isolates were calculated for the overall population in communities as well as LTC facilities. Additionally, monthly ESBL rates were calculated for different genders, age groups, and LHIN groups for the population representing the community only.

### Statistical analysis.

The significance of the differences in ESBL rates between different population groups was calculated by a chi-square test. For segmented time series analysis, the pre-COVID-19 period and COVID-19 period ESBL rates were fit into time series models using the tslm function in R. Autocorrelations of data were tested by a Durbin-Watson test. To control for seasonality, time series models were stratified by two pairs of Fourier sine-cosine terms, and deseasonalized pre- and postintervention time series were created for each population group. Changes in monthly ESBL rates during the COVID-19 period with corresponding confidence intervals and significance values were estimated from the deseasonalized time series by linear regression analysis. Multivariate logistic regression analysis was performed to test for ESBL associations with different demographic and geographic factors using a generalized linear model. All statistical analyses were performed in RStudio version 22.02.1. For all analyses, a *P* value of <0.05 was considered statistically significant.
