# Supplementary material for: Trends in the Rates of Extended-Spectrum-β-Lactamase-Producing Enterobacterales Isolated from Urine Cultures during the COVID-19 Pandemic in Ontario, Canada
Source: Microbiol Spectr. 2023 Jan 16;11(1):e03124-22. doi: 10.1128/spectrum.03124-22 (PMC9927304; doi:10.1128/spectrum.03124-22)
Supplement: Supplemental file 1 — Fig. S1 and S2. Download spectrum.03124-22-s0001.pdf, PDF file, 0.2 MB [file spectrum.03124-22-s0001.pdf]

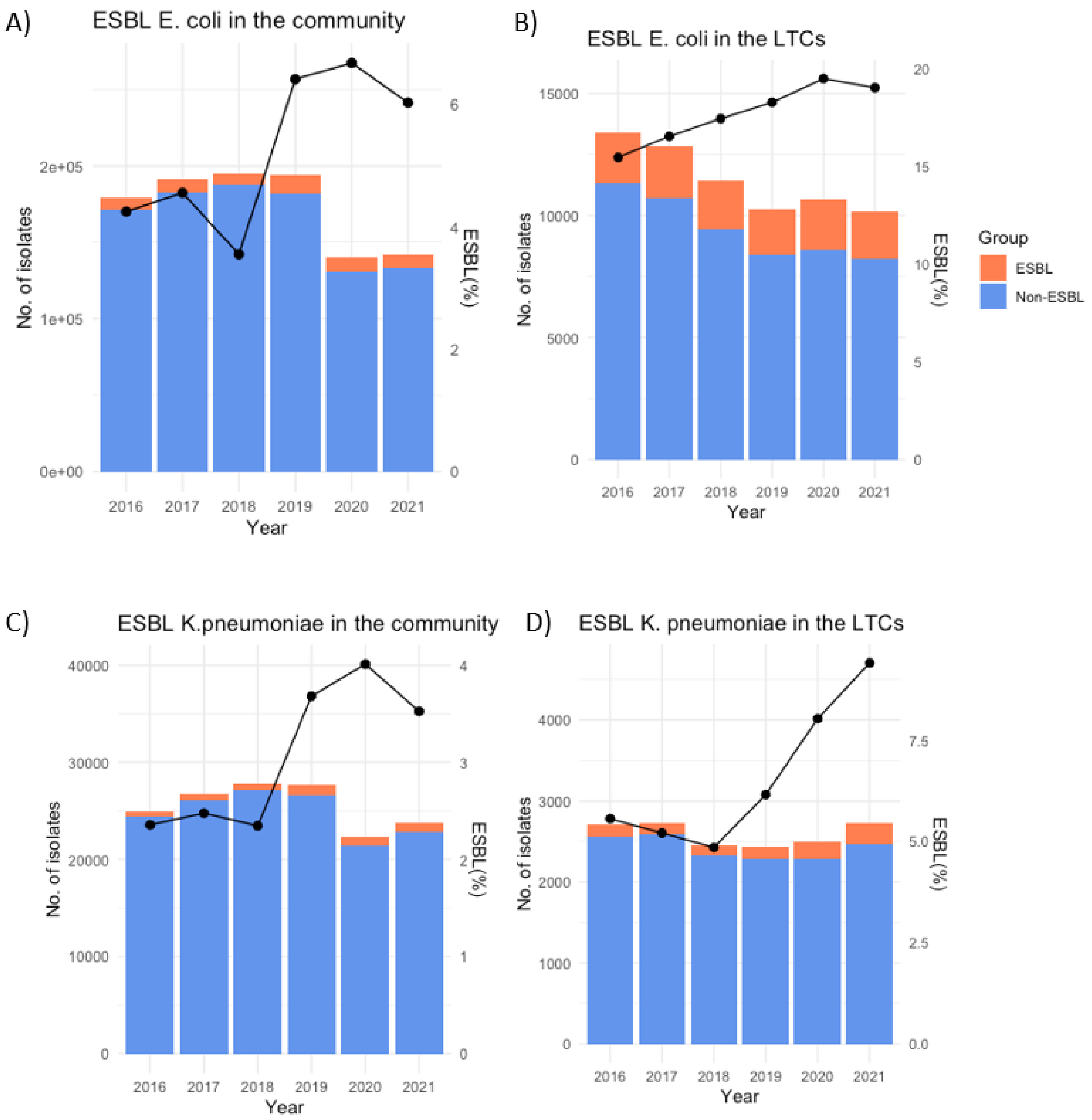

Supplemental figure 1: Numbers and proportions of ESBL producing *E. coli* and *K. pneumoniae* isolated from urine cultures in Ontario, Canada from 2016 to 2021. A) Number and proportions of ESBL and non-ESBL *E. coli* isolated from community acquired urinary tract infections B) Number and proportions of ESBL and non-ESBL *E. coli* isolated from LTC acquired urinary tract infections C) Number and proportions of ESBL and non-ESBL *K. pneumoniae* isolated from community acquired urinary tract infections D) Number and proportions of ESBL and non-ESBL *K. pneumoniae* isolated from LTC acquired urinary tract infections.

A)

B)

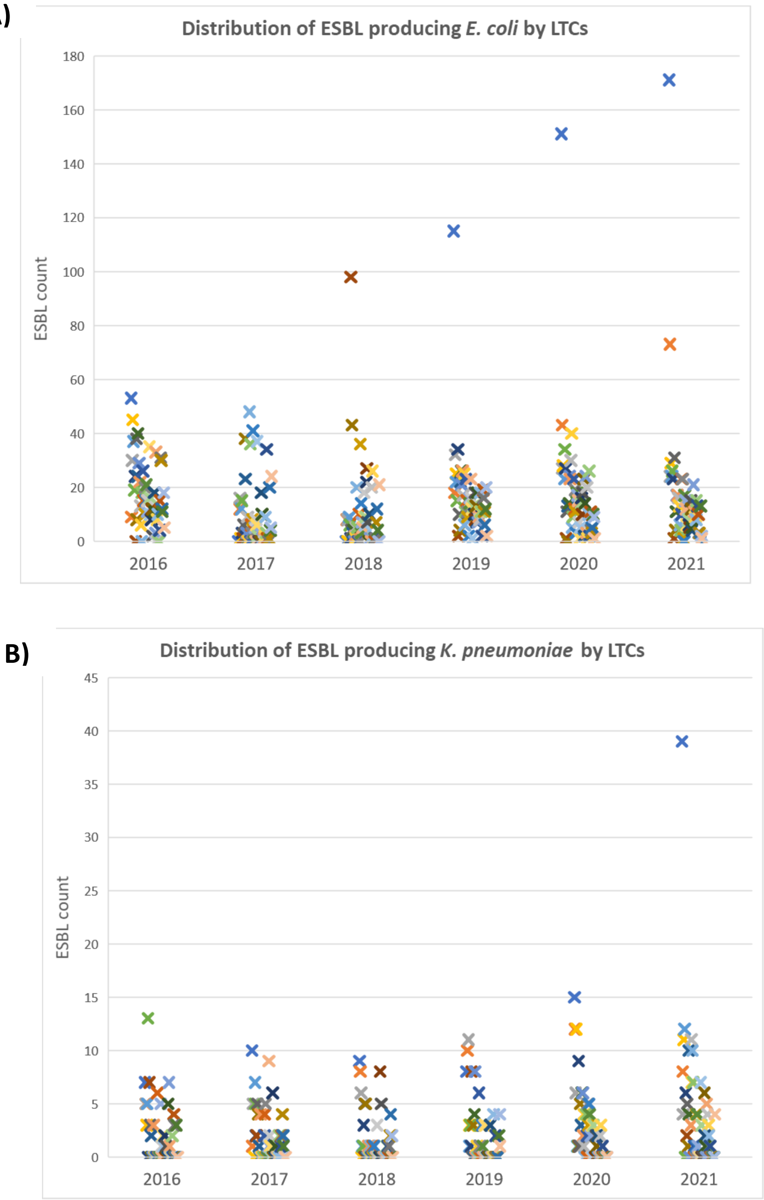

Supplemental figure 2: Distribution of ESBL producing *E. coli* (A) and *K. pneumoniae* (B) isolated from urine cultures from LTCs across Ontario, Canada from 2016 to 2021. ESBL counts for each of the LTCs were plotted against the year of isolation. Data for only top 50 LTCs by ESBL counts were plotted.
